# Evaluation of sparing the pronator quadratus for volar plating of distal radius fractures: a retrospective clinical study

**DOI:** 10.1186/s12891-022-05576-3

**Published:** 2022-06-30

**Authors:** Xiaoxia Huang, Qiyu Jia, Huaqiang Li, Erxat Kerem, Cong Peng, Weiqi Kong, Maimaitiaili Tusunniyazi, Yimurang Hamiti, Dongwei Feng, Yan Zhao

**Affiliations:** 1 Department of Microrepair and Reconstruction, The First Affiliated Hospital of Xinjiang Medical University, Urumqi, Xinjiang China; 2 Department of Spine Surgery, The First Affiliated Hospital of Xinjiang Medical University, Urumqi, Xinjiang China

**Keywords:** Sparing the pronator quadratus, Distal radius fracture, Henry approach, Volar plating

## Abstract

**Background:**

The most commonly used approach for distal radius fractures is the traditional Henry approach. However, it requires an intraoperative incision of the pronator quadratus (PQ) muscle, which results in a series of complications if the repair of the PQ fails.

**Aim:**

The objective of this study was to investigate the efficacy of sparing the pronator quadratus for volar plating of the distal radius fractures.

**Methods:**

Seventy-six patients who suffered from distal radius fractures of types 23-B, 23-C1, and 23-C2 as per the AO Foundation and Orthopaedic Trauma Association (AO/OTA) classification were treated with volar locking plate fixation using either the PQ muscle incision and repair (group A, *n* = 39) or the PQ muscle preservation approach (group B, *n* = 37). Intraoperative index, postoperative efficacy and complications of patients were recorded and evaluated.

**Results:**

All patients were followed up for more than one year after surgery. All fractures achieved union. There were significant differences in mean operative time, mean intraoperative blood loss, and mean fracture healing time between the two groups. Still, there were no significant differences in limb function scores between the two groups at the 12-month postoperative follow-up. Outcomes assessed at 1 week, 1 month, and 3 months after surgery demonstrated significant differences in the mean range of motion and pain-related visual analog scale (VAS) between the two groups. As the range of motion and grip strength increased, the VAS scores decreased, and there was no significant difference between the two groups at 12 months postoperatively. Although tendon irritation and delayed carpal tunnel syndrome were more common in group A than in group B (7.6% vs. 0% and 5.1% vs. 0%, respectively), the differences were not statistically significant.

**Conclusion:**

The modified Henry approach with sparing pronator quadratus muscle has no significant advantage in the range of wrist motion and upper limb function in the late stage. Nevertheless, the intraoperative placement of the plate under the pronator quadratus muscle can shorten the operation time, reduce intraoperative bleeding, reduce early postoperative pain, promote early activity, and improve the patient's quality of life. It is recommended that the pronator be preserved at the time of surgery.

**Supplementary information:**

The online version contains supplementary material available at 10.1186/s12891-022-05576-3.

## Background

Fractures of the distal radius are common and account for approximately 17% of fractures [1]. They are among the most common orthopedic injuries seen in the emergency room [2].This observation is partly due to increased life expectancy, leading to more osteoporotic fractures and an increasing variety of contact sports leading to high energy trauma in the younger population [3].

Volar locking plate fixation has become the standard surgical procedure for treating unstable distal radius fractures [4–6]. Johnson et al. [7] first reported the function of the pronator quadratus muscle to stabilize the distal ulnar radial joint in 1976. The most commonly used approach in distal radius fracture surgery is the traditional Henry approach, which requires slicing open the PQ muscle. There is great controversy regarding the significance of PQ muscle repair and whether the postoperative wrist function and outcomes are affected. Previous studies showed that even in patients with repaired PQ muscle [8], there is still a significant loss of strength in pronation after surgery, which may be related to tissue damage and edema or poor repair. Therefore, this study investigates the patient outcomes following intraoperative PQ muscle preservation with a posteriorly-placed plate.

Clinical data were collected from 76 patients with distal radius fractures who underwent open reduction and plate internal fixation. Patients were grouped based on whether the PQ muscle would be spared during the procedure or not. Intraoperative index, postoperative efficacy, and complications of the patients were evaluated. This retrospective comparative study aimed to investigate the effect of preserving the PQ muscle on wrist function in patients.

## Methods

After the Institutional Review Board's written approval, a retrospective study was conducted between January 2019 and October 2020. 76 patients with distal radius fractures who underwent open reduction and plate internal fixation at the First Affiliated Hospital of Xinjiang Medical University were enrolled in the study. The PQ muscle was cut open in group A, while group B spared the PQ muscle. The inclusion criteria were: (1) age ≥ 18 years; (2) Unilateral displaced and unstable distal radius fractures; (3) Failure of manual reduction; and (4) AO classification 23-B, 23-C1, and 23-C2. The following patients were excluded: (1) ipsilateral or contralateral upper limb fractures and/or dislocation; (2) open fractures; (3) pathological fractures or metabolic bone disease; (4) Fractures greater than 3 weeks in duration; (5) associated nerve or vascular injury requiring repair; (6) Previous history of distal radius fracture on the affected side; (7) mental illness; (8) Poor compliance and lost follow-up.

Before the operation, a panel of experts needed to decide whether the patient should be treated with the PQ muscle sparing technique or the traditional technique. The final decision was then made depending on the actual situation during the operation. The same medical team carried out all surgical procedures. The patient demographics and fracture characteristics are displayed in Table 1. There was no significant difference in the pre-operative variables between the two groups.

**Table 1:** Baseline characteristics of the two groups

	Group A (39)	Group B (37)	*P*
Mean age (years)	56(41, 64)	55(49, 58.5)	0.396
Sex			
Male	17	12	0.317
Female	22	25	
Side of hand			
Left	19	17	0.809
Right	20	20	
AO classification (n)			
B1	9	9	0.871
B2	9	7	
B3	5	8	
C1	8	7	
C2	8	6	
Cause of injury (n)			
Fall injury	23	21	0.871
Falling injury from height	13	14	
Traffic trauma	3	2	
Mean interval from injury to surgery (days)	4(3, 5)	4(3, 5)	0.947

### Surgical procedures

**Fig. 1:**
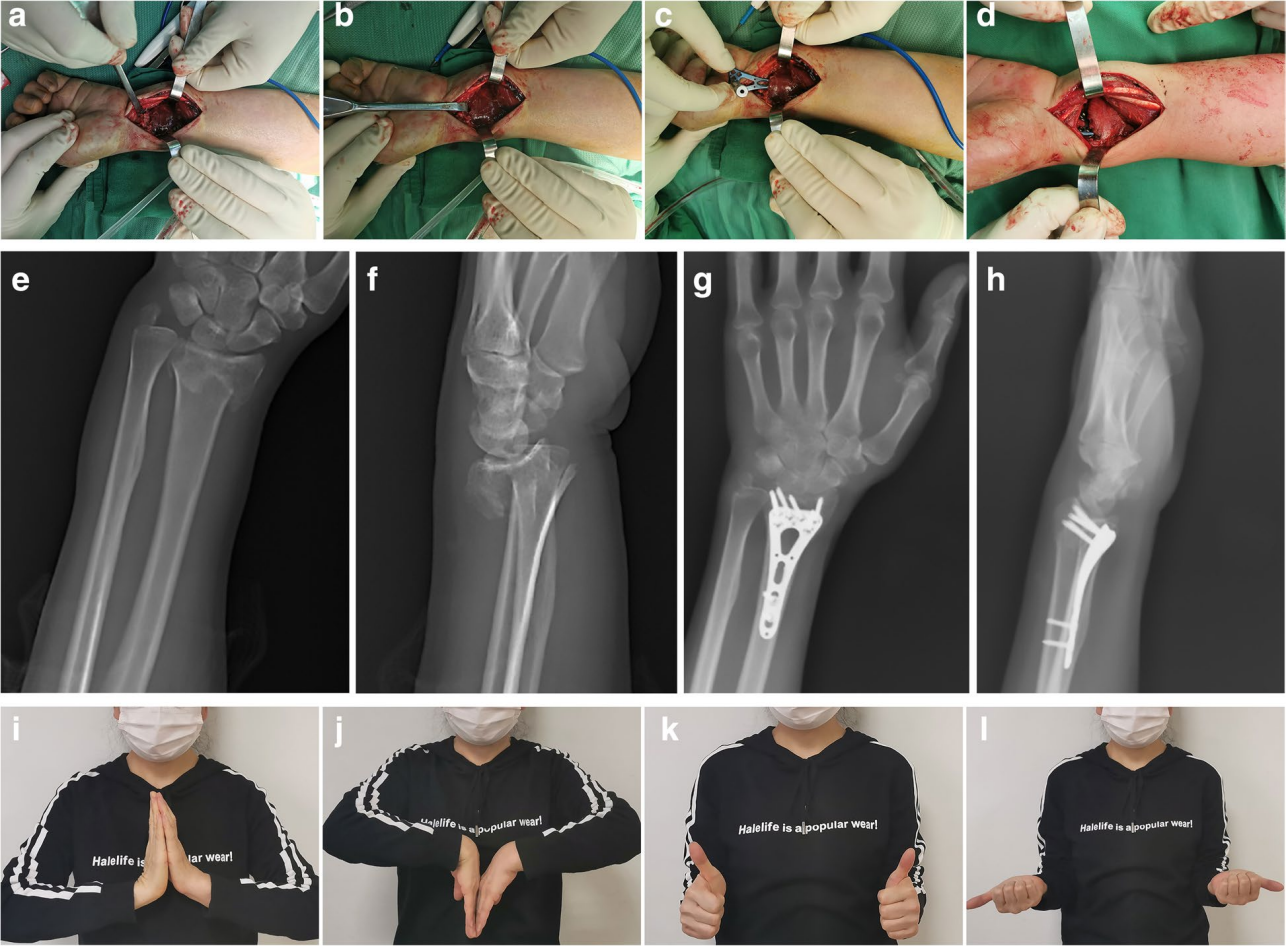
**a** 55-year-old woman with a fall injury resulting in a left distal radius fracture of type B2 according to AO classification. **b** A tunnel was established under the PQ muscle during the operation. **c** The plate was placed below the PQ muscle. **d** Pre-incisional closure macrophotograph. **e–f** Preoperative x-ray of the affected limb. **g-h** Postoperative x-ray of the affected limb. **i-l** Wrist function at 3 months after surgery

**Fig. 2:**
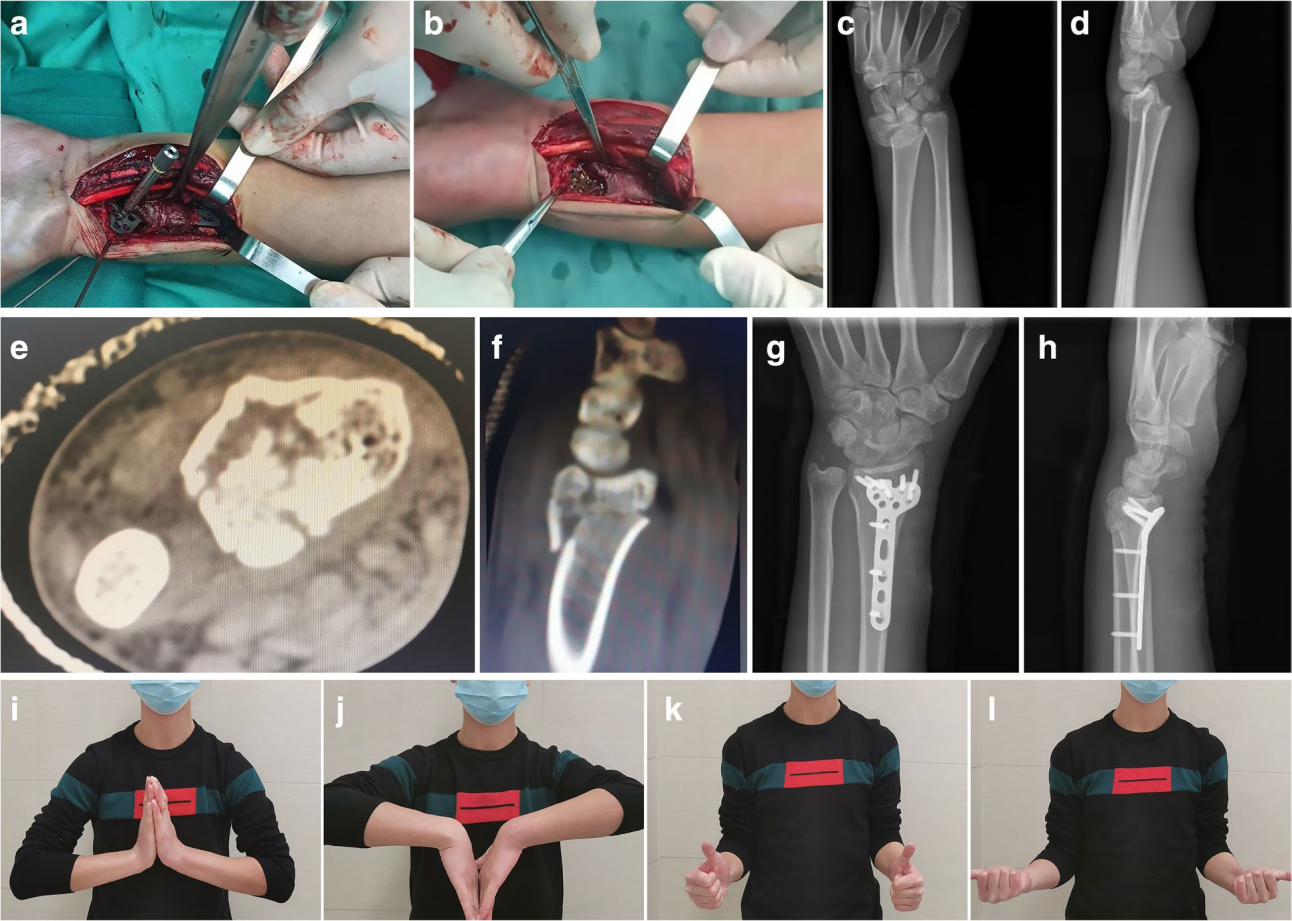
A 21-year-old man with a fall injury resulting in a left distal radius fracture of type C1 according to AO classification. **a, b** Intraoperative sparing of the PQ muscle in the surgical approach. **c-f** Preoperative imaging of the affected limb. **g-h** Postoperative imaging of the affected limb. **i-l** Functional recovery of the wrist joint 3 months after surgery

Patients were placed supine under general or regional anesthesia, and the surgery was performed. The Henry approach through the flexor carpi radialis (FCR) tendon sheath was applied for the Group A patients. The tendon was identified and mobilized to the radial edge of the incision. After incising the FCR tendon sheath, the PQ muscle was exposed and an L-shaped incision was performed along the radial border of the radius to the radial malleolus, and the PQ was then stripped off the radius. After the fragments were repositioned, fluoroscopic confirmation was obtained and a plate was inserted for internal fixation. The PQ was sutured using interrupted 3–0 and 4–0 absorbable sutures. The repair was abandoned if the PQ muscle was severely destroyed or edematous. For patients in Group B, if there was no primary injury to the PQ muscle, the fracture was repositioned without dissecting the PQ muscle by modifying the traditional Henry approach. After completion of the repositioning, a blunt separation between the PQ muscle and the periosteum was performed with a periosteal stripper to establish a tunnel posterior to the PQ muscle, through which the plate was placed. When the fracture was well repositioned and the plate was well positioned with X-ray fluoroscopy, the PQ muscle was bluntly separated with a mosquito clamp to reveal the screw holes and the screws were placed (Figs. 1 and 2).

### Postoperative management

Following the surgery, all patients were on routine prophylactic antibiotics for 3 days and were not immobilized in plaster or brace. Patients were allowed to move their fingers immediately after surgery in both groups. Motion at the wrist joint was initiated 3 weeks post-surgery. At 6 weeks postoperatively, the patient's movement progressed to full active motion tolerated by each patient. Patients were allowed to resume full activity and weight-bearing after radiographic confirmation of fracture healing.

The patients were observed post-operatively for internal fixation loosening and complications such as flexor tendon irritation, traumatic arthritis, joint stiffness and carpal tunnel syndrome. The visual analog scale (VAS) scores of postoperative wrist pain and the forearm rotation angle between the two groups were compared. The Dienst score was used to assess the wrist function, and the imaging indexes (radial height, volar tilt, ulnar inclination) were used to evaluate the efficacy of the surgery.

### Statistical analysis

SPSS Statistics software version 26.0 was used for the statistical analysis. All data were analyzed using a normal distribution test. Mean ± SD was used to represent measurement data with normal distribution. The student's t-test was used to compare measurement data of normal distribution. Conversely, a nonparametric Mann–Whitney U test was applied to compare two groups if the data did not display normal distribution, with data expressed as median and interquartile range. The count variables were analyzed by the Chi-square or Fisher's test, expressed as a number. *P* < 0.05 was considered statistically significant.

## Results

Seventy-six patients who underwent palmar plating for distal radius fractures were enrolled in the study, 39 in Group A and 37 in Group B. All patients were followed up for more than one year after surgery (Figs. 1 and 2). There was a successful union of all fractures. Significant differences were found between the two groups' mean operative time, mean intraoperative blood loss, and mean fracture healing time (*P* = 0. 0, and 0.034, respectively). Still, there were no significant differences in limb function scores between the two groups at the 12-month postoperative follow-up (*P* = 0.362) (Table 2).

**Table 2:** Details of intra- and post-operative variables in the two groups

	Group A (39)	Group B (37)	*P*
Mean operative time (min)	75(70, 80)	55(50, 60)	0.00
The mean operative blood loss(ml)	35(30, 45)	20(20, 25)	0.00
Mean bone union time (weeks)	12(11, 12)	11(11, 12)	0.034
Dienst score (12 months)			
Excellent	23	25	0.362
Good	10	10	
Fair	6	2	

No intraoperative vascular injury wound complications, postoperative re-displacement, tendon rupture, or hardware failure were observed in either group. Moreover, there were no statistically significant differences in the postoperative imaging indexes (radial height, volar tilt, ulnar inclination) compared between the two groups (Table 3). Three patients (7.6%) in the A group suffered tendon irritation (two were not repaired due to severe PQ muscle damage and intraoperative repair difficulties), which was relieved after one year when the plate was removed in stage II. None of the sparing PQ group patients developed flexor tendon irritation after surgery. Two patients (5.1%) in Group A complained of delayed carpal tunnel syndrome.

**Table 3:** Postoperative imaging indexes

	3 days			1 month			3 months		
Group	A	B	*P*	A	B	*P*	A	B	*P*
Radial height	11.36 (11.30, 12.55)	12.14 (11.43, 12.55)	0.451	11.80 (11.31, 12.56)	12.20 (11.45, 12.50)	0.673	11.80 (11.20, 12.58)	12.10 (11.40, 12.25)	0.480
Volar tilt	12.50 (11.60, 13.20)	12.25 (11.50, 12.65)	0.095	12.40 (11.80, 13.30)	12.16 (11.60, 12.65)	0.100	12.50 (11.90, 13.30)	12.30 (11.50, 12.64)	0.143
Ulnar inclination	23.10 (22.60, 23.60)	23.50 (22.91, 23.70)	0.260	23.30 (22.58, 23.60)	23.46 (22.88, 23.65)	0.188	23.20 (22.60, 23.56)	23.55 (22.91, 23.60)	0.252

**Fig. 3:**
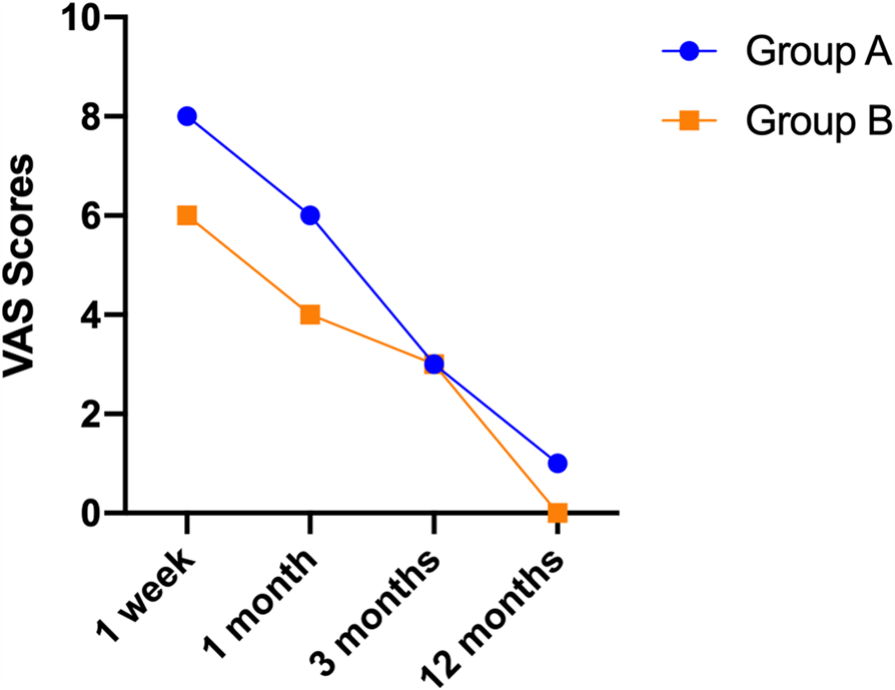
One-year trend in VAS scores for groups **A** and **B**

**Fig. 4:**
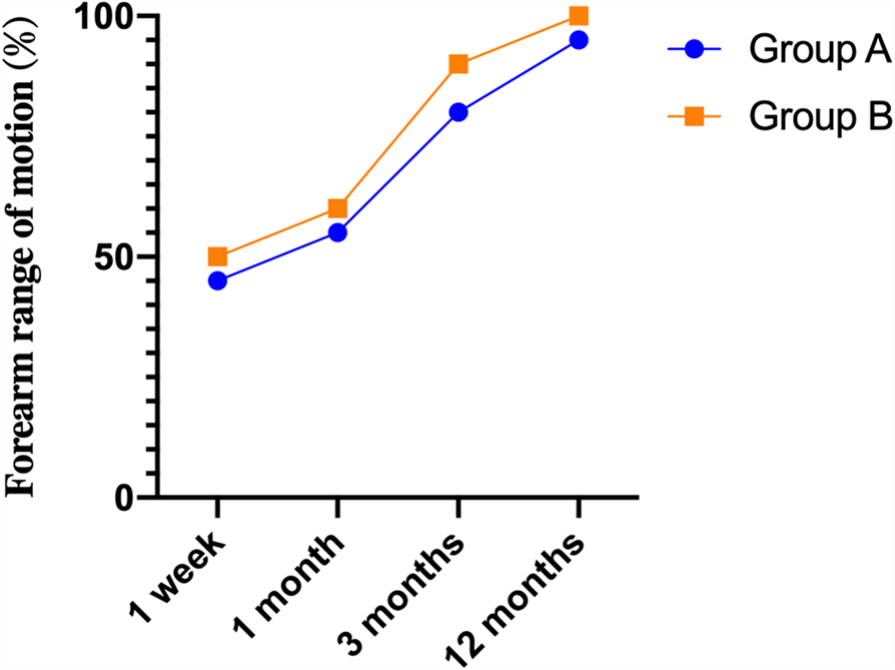
One-year trends in forearm range of motion for group **A** and **B**

The range of motion measurements for each interval is shown in Fig. 4. Outcomes assessed at 1 week, 1 month, and 3 months after surgery demonstrated significant differences in the mean range of motion and pain-related VAS scores between the two groups. The mean values for all variables gradually improved over the year as the range of motion and grip increased and VAS scores decreased (Figs. 3, 4). There was no significant difference between the two groups at 12 months postoperatively.

## Discussion

There is controversy about whether to spare the PQ muscle intraoperatively in patients with distal radius fractures [9–13]. Even the repair of the PQ muscle after fracture reduction remains a topic of debate in the current literature. Some surgeons, including Goorens CK, believe that repairing the PQ muscle only relieves the patient's postoperative pain at an early stage [14, 15]. In addition, some surgeons consider that whether or not the PQ muscle is repaired intraoperatively is not significantly related to the patient's postoperative functional recovery of the wrist [16, 17]. Fenglei Shi concluded in a study that whether or not to repair the PQ muscle may not improve late wrist function in patients [18]. However, Chun-Kuan Lu suggested that repairing the PQ muscle may have some effects on pronation strength, and it is uncertain whether repairing the PQ is a protective factor in preventing flexor tendon complications [19].

Despite these statements, 83% of American hand surgeons repair the PQ muscle following volar plate fixation [20]. However, sometimes we are faced with the situation that the PQ muscle is hard to restore. We found that intraoperatively, the placement of the plate and the suturing of the PQ muscle were often difficult due to muscle tissue lesion, bleeding, and edema following incision of the PQ muscle. A clinical case observation study by Swigart et al. revealed that the failure rate of PQ muscle repair was 4%, with 1 in 24 patients failing [21]. It is now being proposed that maximum preservation of the PQ muscle can decrease intraoperative bleeding to enhance the repairable anterior rotator muscle [22, 23]. At the same time, some scholars recommend sparing the PQ muscle [24, 25] or completely repairing the PQ muscle at the end of surgery [25]. However, it is generally difficult to repair the PQ muscle at the radial margin of the radius because the muscle fascia is not strong enough to hold the sutures in place. Many recent studies have demonstrated that complete sparing of the PQ muscle relieved postoperative pain, improved rotational function, and facilitated the early functional recovery of the patient's wrist [26–29]. Similarly, this study confirmed that among 76 patients with unstable distal radius fractures, the 37 cases undergoing PQ muscle-sparing surgery demonstrated shorter operative time, less bleeding, fewer complications, less postoperative pain, and early functional wrist exercise, which facilitated the early functional recovery of the affected limb.

The use of volar locking plates for distal radius fractures has become increasingly popular in the last few years [30, 31]. Several studies have reported that patients achieve better functional scores and fewer complications postoperatively compared to other surgical techniques [11, 30, 31]. Despite the benefits of volar plating, complications are common, especially in the flexor tendons of the fingers and thumb (tenosynovitis, adhesions, ruptures) due to mechanical irritation between the volar plating and the tendons [32–34]. This technique can diminish these complications since the PQ muscle protects the flexor tendon and allows space for tendon gliding.

Jung et al. [35] measured the anatomy of the cadaveric specimen with CT imaging and concluded that the distal fracture of the radius was fixed using a small incision approach. The distal fracture fragment was large enough to place the distal row of screws without opening the PQ muscle, and the plate was appropriately placed. The PQ muscle covering the implant's surface prevents risks such as tendon abrasion, which indicates that the role of the PQ muscle in sheathing the implant cannot be ignored.

Carpal tunnel syndrome is a severe complication after surgical treatment of distal radius fractures [36–38]. Kashir et al. [23] first described an approach for splitting the brachioradialis muscle. By following this approach, they concluded that the integrity of the PQ was preserved, and all distal radius fractures requiring a volar plating could be treated without further incision. They reported no nerve injury or subluxation. However, it was found that this approach involves the radial artery during surgery, and the latter is highly susceptible to damage. Chul ki Goorens et al. obtained satisfactory results with the Minimally Invasive Pronator Quadratus Sparing Approach (MIPO) for distal radius fractures. However, this technique is more demanding for the surgeon [39], and the incision used in the MIPO technology for distal radius fractures are adjacent to the median nerve throughout the operation, and unfamiliarity with the procedure, repeated pulling of the incision, and placement of screws during the surgery may easily irritate the median nerve and cause nerve injury. Therefore, it was believed that adequate surgical field exposure is a prerequisite to avoid complications [27, 29].

The typical anatomical structure of the PQ muscle is the basis of its function [40]. It was found that the PQ muscles were heavily scarred, significantly atrophied, and adhered to the surrounding tissue after suturing. Muscle function is mainly impaired due to the distortion of its anatomy. This impairment is probably due to the disruption of the blood supply from the radial artery to the muscle after the latter has been severed. Hence, affecting the healing of the muscles.

Moreover, the PQ muscle is also a vital blood supply to the periosteum, with its provision from the anterior interosseous artery and the PQ muscle branches of the radial and ulnar arteries, as well as the posterior interosseous artery playing a crucial role in the healing of distal radius fractures. In this study, the mean bone healing time in the sparing PQ group was 11 weeks, lower than the 12 weeks in the conventional incision group. In addition, PQ muscle is a brittle piece of muscle, which is challenging to suture and often tears apart after suturing. The local soft tissue tension increases significantly, especially after the insertion of the plate, making it difficult to pull the muscle together.

Many studies have been reported on the treatment of distal radius fractures with various sparing of the PQ muscle, most of which analyzed the patients' near future postoperative functional recovery of the wrist. However, there are fewer studies on the long-term forearm rotation angles. In this study, we compared the long-term postoperative forearm and wrist function between two groups of patients with or without complete preservation of the anterior rotator muscle, and also focused on the postoperative pain scores of the patients. Satisfactory fracture reduction and good wrist function can be obtained with the palmar approach no matter whether the PQ muscle is preserved or not. Improved patient outcomes are observed when the respective surgical indications are mastered, and the articular surface reduction is adequate. However, preserving the PQ muscle to rehabilitate the forearm rotation is more advantageous in the short term.

It was previously believed that the PQ muscle was less critical, and that the anterior rotator function was limited; however, this is not the case. Jesper Sonntag et al. [41] concluded in a study that with or without repair of the PQ muscle after incision, ultrasound results showed that both were shorter than the healthy side. Still, the shortening was more significant in the unrepaired group. The function of the PQ muscle depends on its normal structure, and it is believed that incision of the PQ muscle with or without repair produces scarring that further affects forearm rotation function. Early forearm rotation is likely to cause a re-tear of the PQ muscle, leading to poor forearm function recovery. Therefore, it is recommended to avoid excessive forearm rotation for 3 weeks after surgery and wait for the scar repair of the PQ muscle to stabilize before active exercise, which is one of the reasons for the poor recovery of early forearm rotation. Nevertheless, it does not mean that the forearm rotation function is significantly limited at the later stage when the PQ muscle is dissected intraoperatively. This study showed no significant difference in the ROM of forearm rotation between the two groups at 12 months postoperatively. Hence, the sparing of the PQ muscle intraoperatively did not demonstrate a significant advantage in the long-term forearm rotation function. However, performing PQ muscle preservation is strongly recommended as it decreases the risk of muscle re-tear in rehabilitation, dramatically reduces the patient's pain, and encourages exercise.

In this surgical technique, it was found that bleeding before skin closure and after the release of the tourniquet were often minimal and sometimes did not even require any postoperative drainage system. It can be explained by the intact PQ muscle and by the cushioning effect of the muscle on the volar plate. The short duration of the procedure significantly decreases the risk of iatrogenic infection.

The AO classification and Fernandez classification are used to classify distal radius fractures, and the AO classification is more popular to guide treatment and determine prognosis. This study included the AO classification of distal radius fractures type B and C1 and C2. In addition, all types of fractures of the distal radius are generally treatable with a volar plate. However, in the case of type A fractures of extra-articular fractures, in order to avoid disruption of the blood supply and to reduce the burden of surgery on the patient, type A fractures are recommended to be treated with manual reduction and plaster fixation or external fixation combined with Kirschner wire since the fracture line is located at the level of the PQ muscle and the PQ muscle itself is a partial injury preoperatively. For comminuted fractures, a fragment-specific plate can be used for fixation.

Furthermore, the following data were collected from the surgical technique of PQ muscle locking plate preservation in the treatment of distal radius fractures. (1) The distal incision should not exceed the watershed line of the distal radius. Traumatic arthritis causes severe discomfort. (2) For dorsally displaced fracture blocks, especially AO fracture type B, reduction and fixation may be assisted by Kirschner wire or small dorsal incisions. (3) Intraoperative temporary fixation of Kirschner wire should be removed promptly after the insertion of 3–4 locking screws to avoid interfering with the implantation of other screws. (4) The implanted screws should not be too long to prevent damage to the dorsal extensor tendon. The ‘carpal shoot-through view’ can be used to determine whether the screws fixing the metaphysis have penetrated the carpal joint cavity [42]. (5) Repeated muscle traction during the procedure should be avoided to prevent secondary injury to nerves, blood vessels, and tendons. (6) Maintaining the wrist in the flexed position during the procedure facilitates reduction and fixation. (7) This approach does not reveal the carpal cavity, and further management of the cavity requires a combination of arthroscopic carpal techniques. (8) A possible drawback of sparing the pronator quadratus is the same as that of MIPO [39], both of which lack direct visualization of the fracture reduction. If intraoperative exposure and fixation reduction are problematic, traditional surgical fixation should be chosen as soon as possible.

With the development of the modern economy and the increasing aging population, the proportion of distal radius fractures presenting in an emergency is high; and people's demand for quality of life is increasing. The collective goal that doctors and patients pursue is achieving optimal recovery, reducing pain, and returning to society early with minimal trauma and tissue damage. In this study, the efficacy of sparing the PQ muscle in treating unstable distal radius fractures was investigated. It concluded that sparing the PQ muscle with a modified Henry approach is feasible for treating intra-articular fractures. The physiological structure of the PQ muscle is preserved, and the contact between the plate and the tendon and nerve is better isolated, which avoids stimulation of the nerve and tendon and reduces the possibility of tendon abrasion. Furthermore, the risk of tissue bleeding and edema after incision of the PQ muscle and eventually adhesions with the surrounding tissue is avoided. The disadvantage is that sparing the PQ muscle may complicate fracture reduction. It then requires fixation with the aid of multiple Kirschner wires or a small dorsal incision. It is not very operable for AO-type C3 distal radius fractures with serious comminution.

Several limitations existed in this study. First, its retrospective study design and the possibility of selection bias. Second, this was a single-center study that enrolled only a small number of patients. High-quality randomized controlled trials with a larger sample size are still needed to reinforce these results. Third, this study did not include distal radius C3 type fracture cases. A larger sample size containing more fracture patterns would be helpful in a future study.

## Conclusion

The modified Henry approach with sparing pronator quadratus muscle has no significant advantage in the range of wrist motion and upper limb function in the long term. However, the intraoperative placement of the plate under the pronator quadratus muscle can shorten the operation time, reduce intraoperative bleeding, may reduce early postoperative pain, promote early activity, and improve patients' quality of life. It is recommended that the pronator be preserved at the time of surgery.

## Supplementary information

**Additional file 1.**

## Data Availability

The datasets analyzed during the current study are available from the corresponding author on reasonable request.

## Abbreviations

PQ: Pronator quadratus
FCR: The flexor carpi radialis
VAS: The visual analogue scale scores
MIPO: Minimally Invasive Plate Osteosynthesis
ROM: Range of movement
